# Migrants’ perceptions of health system responsiveness and satisfaction with health workers in a South African Province

**DOI:** 10.1080/16549716.2020.1850058

**Published:** 2020-12-14

**Authors:** Janine A. White, Jonathan Levin, Laetitia C. Rispel

**Affiliations:** a School of Public Health, Faculty of Health Sciences, University of the Witwatersrand, Johannesburg, South Africa; b Centre for Health Policy & South African Research Chair, School of Public Health, Faculty of Health Sciences, University of the Witwatersrand, Johannesburg, South Africa

**Keywords:** Migrants, health care, health system responsiveness, patient satisfaction, south Africa

## Abstract

**Background**: There is global emphasis on quality universal health coverage (UHC) that is responsive to the needs of vulnerable communities, such as migrants.

**Objective**: Examine the perceptions of migrants on health system responsiveness (HSR) and their satisfaction with health workers in public health facilities of a South African Province.

**Method**: We conducted a cross-sectional study in 13 public health facilities. Following informed consent, we used a semi-structured questionnaire to collect sociodemographic information, patient perceptions of HSR and their satisfaction with health workers. Two open-ended questions gave patients the opportunity to comment on the health facility visit. We applied descriptive and multivariate analyses to our data, and thematic analysis to the qualitative responses.

**Results**: A total of 251 migrant patients participated in the study, giving a response rate of 80.7%. The majority of patients were female (81.1%), and the mean age was 31.4 years. 30.0% of patients reported that they waited too long; 94.3% that the consulting nurse or doctor listened to them; and 89.4% that they received information about their condition. However, 81.7% said they did not know the name of the consulting nurse or doctor. The mean scores on patients’ satisfaction with health workers ranged from 7.0 (95% CI 6.42–7.63) for clerks, 7.7 (95% CI 7.4–8.0) for security guards, 7.4 (95% CI 7.1–7.6) for nurses and 8.3 (95% CI 7.93–8.63) for doctors. The predictors of patient satisfaction with nurses were being given information about their condition; polite treatment, time spent in facility, whether they received prescribed medicines; and stating that they would refer the health facility to family/friends. Four overlapping themes emerged: health workers’ attitudes; time waited at the health facility, communication difficulties; and sub-optimal procedures in the health facility.

**Conclusion**: UHC policies should incorporate migrant patients’ perceptions of HSR and the determinants of their satisfaction with health workers.

## Background

Amidst the global discourse on quality universal health coverage (UHC), the world faces an unprecedented migrant crisis [1]. In 2019, an estimated 70 million people were displaced, the majority in low and middle-income countries [1]. Migrants refer to individuals who have moved across an international border, regardless of legal status, the reasons for the movement and/or whether the movement is voluntary or involuntary [2]. Hence, refugees are included in this definition [2]. However, migrants are often excluded from or adversely included in UHC reforms, as was found in a review on the inclusion of migrants in the UHC systems of Indonesia, Malaysia, Philippines, Singapore, and Thailand [3].

Concomitant with the global emphasis on UHC is the recognition that high-quality health systems are linked inextricably to the achievement of such UHC [4]. The 2018 Lancet Global Commission on high-quality health systems highlighted the vulnerability of migrants to poor-quality health care, and recommended research on health system responsiveness (HSR) and research that incorporates patient voices and experiences [5]. HSR is defined as the ‘responsiveness of health systems to the legitimate expectations of populations regarding how they are treated’ [6]: p.77. HSR has assumed increasing importance since the seminal 2000 World Health Report [7], which underscored the importance of the performance of health systems. HSR focuses on the non-clinical aspects of the quality of the health system (e.g. waiting times and health provider attitudes) [8], and is important for patient satisfaction with the health care system [9]. Patient satisfaction is an important indicator of the quality of care provided, particularly for vulnerable groups such as migrants, who may be at risk of health inequity and social exclusion [10]. In addition, evidence suggests that there is a positive correlation between patient satisfaction and health-seeking behaviour [6].

Health care utilisation studies among migrant patients are useful to get an indication of their perceptions of the non-clinical aspects of the quality of the health system (i.e. HSR), their satisfaction with, and their experiences of the health system [11]. In high-income countries, several studies have described the experiences and perceptions of migrants of the health care systems in their host countries [12–20]. A 2010 review of migrants using health care services in high-income countries demonstrated negative associations between patient satisfaction scores and gender, age and education levels [10]. Low patient satisfaction scores were also associated with poor communication, lack of information, and disrespectful interactions with health care providers [10].

Although Canada is known for its inclusive immigration and refugee policies, a 2016 review found that there were cultural, communication, and healthcare system barriers to immigrants’ access to primary healthcare (PHC) services [12]. Other studies in the EU found that undocumented migrants underutilised healthcare services, and/or received sub-optimal quality health care, and this was linked to their awareness of their entitlements [14]. In the UK (UK), studies have found that gypsies, travellers and minority ethnic groups experienced high levels of inequalities in the access and/or the quality of care they received in the public healthcare system [15,16]. In Germany [17,18] and Denmark [19], studies found that migrant patients’ satisfaction with health care services were influenced by communication by, and cultural sensitivity of, health care providers. A 2016 qualitative study that explored HIV-positive migrants’ experiences in the Swedish health care system found that they appreciated access to free antiretroviral therapy, but felt discrimination in health care settings outside of the infectious diseases clinics [21].

Similarly, studies in Africa have highlighted complex and at times contradictory experiences of migrants in the public health sector [22–25]. In Kenya, a study found that both Kenyans, migrants and refugees had similar health service utilisation experiences, but migrants and refugees experienced discrimination as well as language and cost barriers [22]. In Botswana, a 2010 study found that Zimbabwean migrants experienced cost and language barriers, lack of choice of medical practitioner, negative attitudes of medical staff, and fear of police or immigration officials [23]. In Ethiopia, a study among Eritrean refugees in refugee camps found that they had concerns about the limited health care facilities and their inability to access essential health care services [24].

In South Africa, the Constitution states that all people, regardless of citizenship, have the right of access to health care services, and that no one may be refused emergency medical treatment [26]. However, migrants’ access to the South African healthcare system is a complex matter. There are contradictions and/or confusion between laws and policies, different interpretations of laws or policy at health facility level [27] and a policy vacuum on migration, health, and migrants’ needs [28]. Furthermore, South Africa’s public healthcare system struggles to provide high-quality care to people, regardless of their nationality or refugee status [29]. Nonetheless, studies have documented the health care access and/or utilisation barriers faced by migrants or refugees [25,30], exacerbated by lack of legal documents [31], xenophobia and insensitive health workers [32–34]. However, many of these studies are small case studies [25,30–32], with a dearth of quantitative studies on migrant patient satisfaction and their perceptions of HSR.

Importantly, South Africa has committed itself to major health sector reforms, the most prominent of which is the implementation of the National Health Insurance (NHI) system. The latter is a health financing reform intended to pool funds to provide access to quality health services and to reform the health system towards the achievement of UHC [35]. HSR is a critical goal of national health systems [11] and thus, remains important in the realisation of the NHI in South Africa.

In light of the global imperative of quality UHC, we conducted the study to contribute to, and shape, the discourse on quality, and inclusive UHC in South Africa. The overall aim of this study was to examine the perceptions of migrants on health system responsiveness (HSR) and their satisfaction with health workers in public health facilities of a South African Province.

## Methods

### Study setting and facility sampling

The setting for this cross-sectional study was the public health care system of the Gauteng Province in South Africa. In 2018, Gauteng had an estimated 419 169 international migrants and is reportedly the province with the largest proportion of migrants in South Africa [36].

The public health care system in Gauteng consists of four central, academic referral hospitals, three regional tertiary hospitals, nine regional hospitals, 11 district hospitals, 30 community health centres, and 290 primary health care clinics, and six specialised psychiatric and tuberculosis hospitals [37].

We used a two-stage cluster sampling approach. We selected two facilities randomly from each of the following categories: central hospital; regional hospital; regional tertiary hospital; district hospital; community health centre; and primary health care clinic. There is only one mother and child hospital in the province; hence, we sampled 13 public health care facilities in Gauteng. Specialised psychiatric and tuberculosis hospitals were excluded because these do not provide ambulatory care services.

### Study population

The primary population of interest was migrants using ambulatory care services in public health facilities in Gauteng Province. The eligibility criteria were: an international adult migrant, over the age of 18; seeking ambulatory care; and providing voluntary, informed, written consent to participate in the study. All medical and surgical emergencies were excluded.

### Measures

We designed a semi-structured questionnaire in English. The questionnaire consisted of a socio-demographic section (7 questions); experience of the health care consultation and perceptions of HSR (8 items); patients’ assessment of their interaction with health workers (5 items).

The section on socio-demographics elicited information on age, gender, education, country of origin, and length of time in South Africa. The section on patients’ experiences of the health care consultation incorporated proxy questions on HSR, specifically the domains of prompt attention, communication, confidentiality, dignity, and quality. The total time spent in the health facility was a proxy for the domain of prompt attention, and was measured on a 3-point scale of too long (3), just right (2) or too short (1). The domains of communication, confidentiality, dignity, and quality were measured on a dichotomous scale of yes or no. The domain of communication was measured by three questions: whether the patients knew the name of the health care provider, whether the provider listened to them; and whether they received information on their condition. The domain of confidentiality and dignity were measured on whether their privacy was respected and whether they were treated politely respectively. The domain on quality was measured on whether patients received their prescribed medicine and whether they would refer a sick friend or family member to that particular health facility.

The section on patients’ interactions with health workers requested patients to rate the service received from different health worker categories on a scale from 1 (very unhappy) to 10 (very happy). These categories were clerks, doctors, nurses, and security guards. When patients did not encounter the service of a health care worker, they were asked to indicate ‘not applicable’.

Two open-ended questions elicited the patients’ opinions on their health care visit, HSR and/or quality and their suggestions for health care or quality improvement.

### Piloting

Prior to data collection, five health system researchers assessed the content validity of the questionnaire. We piloted the questionnaire at two non-sampled health facilities to determine the clarity of questions, the need for possible adjustments, and the time to complete the questionnaire. We made minor adjustments to the questionnaire to improve clarity of the items asked, and excluded the pilot from the main study results. Cronbach’s alpha (CA) for the pilot data was 0.63. This score indicates reasonably good reliability as evidenced by the inter-item correlation.

### Data collection

During 2018, fieldwork was conducted on three randomly selected days at each facility. In the case of PHC clinics, we selected three days randomly between Monday and Friday. In the case of community health centres and hospitals, we selected two days randomly between Monday and Friday, and one day randomly on the weekend.

The principal investigator (JW) recruited and trained five fieldworkers to assist with the study. On the fieldwork days, all eligible patients were approached after their health care consultation and upon exiting the relevant facility. Potential participants were informed that participation was voluntary, and that they could withdraw from taking part at any point, without prejudice or negative consequences. They were also reassured of the confidentiality of the study, and that no member of the research team was part of the health care authorities.

Following informed consent, a member of the research team administered the questionnaire using a tablet, with direct data entry into Research Electronic Data Capture (REDCap), a secure, web-based application designed to support data capture for research studies [38]. The principal researcher checked each questionnaire for completeness.

### Statistical analysis

We computed descriptive statistics on socio-demographic characteristics and migrant patients’ perceptions of HSR and their satisfaction with health workers. For descriptive purposes we categorized both age and number of years living in South Africa. The independent variables included socio-demographic characteristics i.e. age, gender, highest qualification attained, number of years living in South Africa, and the type of facility utilised e.g. clinic or hospital. We examined the HSR domains (communication, confidentiality, dignity, prompt attention and quality) as both response and explanatory variables.

In the inferential statistics, all variables found to be statistically significant at a 20% level in the unadjusted models were included in the final multiple logistic regression (e.g. did the doctor/nurse listen to you) or ordinal logistic regression (e.g. amount of time spent on the visit). We examined the factors associated with patients’ perceptions of HSR, and the relationship between HSR and patient satisfaction with health workers in the multiple logistic and ordinal logistic regression models. For the multiple logistic and ordinal logistic regression analysis, we grouped the 13 health care facilities into three categories: central and regional tertiary hospitals were combined into one category; district and regional hospitals into another; and PHC clinics and community health centres into one category of PHC facilities. We used STATA® 15 for analysis.

### Analysis of qualitative comments

We analysed the qualitative comments data using thematic analysis [39]. The analysis was an iterative process beginning with reading and re-reading the comments, before coding the comments. JW and LR read and coded all the comments independently and inductively, i.e. using the participants’ own words. These inductive codes were then grouped into broader themes. JW and LR reached consensus on the themes, thereby establishing inter-coder agreement. Once the themes were agreed, JW re-examined the data, and categorised the responses into these themes.

### Ethical considerations

The Human Research Ethics Committee (Medical) of the University of the Witwatersrand provided ethical approval for the study (#: M170988). We also obtained permission from the Gauteng Provincial Department of Health (DoH), the district health authorities, and the managers in charge of each of the selected health facilities. We adhered to all ethical procedures during data collection. We provided both written and verbal explanation of the study to all study participants. We only proceeded with the study following study participants’ informed, written consent. We adapted an existing distress protocol in the event of negative reactions by the study participants during the interview, with clear referral procedures for assistance [40]. The purpose of the distress protocol was to manage any distress from patients that may arise during data collection, in case any question served as a trigger of a previous negative or traumatic experience in a health facility.

## Results

### Socio-demographic and patient characteristics

We invited 311 migrant patients across the 13 health care facilities to participate in the study: 251 consented to study participation, translating into a response rate of 80.7%. The majority of participants were female (81.1%), living together/married (69.9%), and from Zimbabwe (55.7%) (Table 1). The mean age of participants was 31.4 years. The majority of participants were recruited at PHC facilities (57.1%) (Table 1). The mean number of years living in South Africa was 6.5 years.

**Table 1.** Socio-demographic and patient characteristics of the study sample

Variable	n (%)
**Age category**	
<25 years	59 (24.5)
25–34 years	104 (43.1)
35+	78 (32.4)
**Gender**	
Female	202 (81.1)
Male	47 (18.9)
**Marital status**	
Single/Divorced/Separated/Widowed	75 (30.1)
Living together/Married	174 (69.9)
**Place of birth**	
East Africa	16 (6.5)
Burundi	3 (1.2)
Ethiopia	3 (1.2)
Kenya	2 (0.8)
Somalia	6 (2.4)
Tanzania	1 (0.4)
Uganda	1 (0.4)
Central Africa	21 (8.7)
Cameroon	1 (0.4)
Democratic Republic of the Congo	20 (8.3)
North Africa	1 (0.4)
Sudan	1 (0.4)
Southern Africa	194 (78.9)
Lesotho	7 (2.8)
Malawi	28 (11.4)
Mozambique	17 (6.9)
Zambia	5 (2.0)
Zimbabwe	137 (55.7)
West Africa	13 (5.3)
Ghana	3 (1.2)
Ivory Coast	1 (0.4)
Nigeria	9 (3.7)
Outside African region: Pakistan	1 (0.4)
**Highest qualification**	
None/Primary	35 (14.1)
Secondary School	167 (67.1)
Post-secondary/Diploma/Higher	47 (18.9)
**Number of years living in South Africa**	
< 2 years	32 (13.0)
2–9 years	148 (60.2)
>10 years	66 (26.8)
**Patient distribution across health care facility**	
Central/Regional Tertiary hospital	63 (25.5)
District/Regional hospital	43 (17.4)
PHC facility	141 (57.1)

### Patients’ perceptions of responsiveness

**Figure 1.**
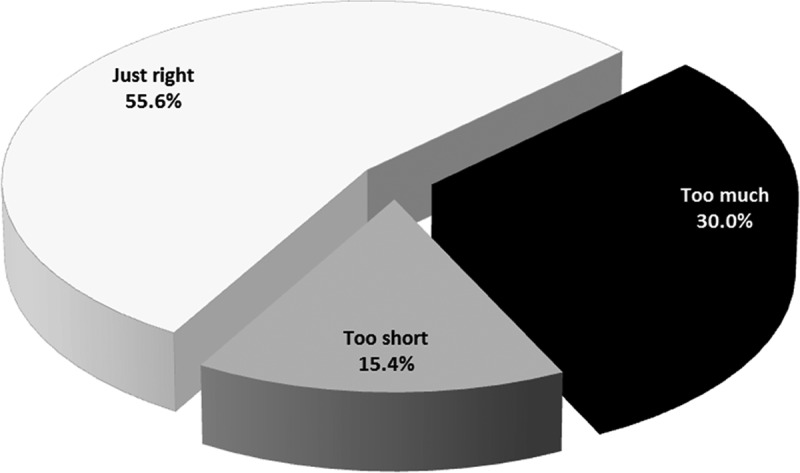
Patient perceptions of time spent in the facility

In the domain of prompt attention, 55.6% reported that the amount of time for their visit was ‘just right’, but almost one-third (30.0%) reported that they waited too long (Figure 1).

In the domain of communication, the majority of patients (94.3%) reported that the consulting nurse or doctor listened to them; and 89.4% reported that they received information about their condition. However, 81.7% of patients reported that they did not know the name of the consulting nurse or doctor. For the domain of confidentiality, 93.5% of participants reported that their privacy was respected, while 92.3% of patients indicated that they were treated politely (dignity domain).

Within the domain of quality, the majority of participants reported that they received their prescribed medication (80.6%); and 85.0% of patients indicated that they would refer a sick friend or family member to the facility.

### Patients’ satisfaction with health workers

The mean scores on patients’ satisfaction with the interaction with health workers ranged from a mean score of 7.0 (95% CI 6.42–7.63) for clerks, 7.7 (95% CI 7.4–8.0) for security guards, 7.4 (95% CI 7.1–7.6) for nurses and 8.3 (95% CI 7.93–8.63) for doctors.

### Factors influencing patients’ perceptions of responsiveness

Table 2(a–d) shows the HSR domain results from the multiple logistic and ordinal logistic regression models.

**Table 2a.** Multivariate logistic regression model of socio-demographic and facility factors influencing patients’ perceptions of responsiveness (by domain: communication)

		Domain: Communication					
		Do you think the nurse/doctor listened to you?		Do you know the name of the nurse/doctor who attended to you?		Were you given information about your condition?	
Variable		OR	p-value/ CI	OR	p-value/ CI	OR	p-value/ CI
Age						1.07	p = 0.159
							0.97–1.19
Place of birth	Reference: North Africa/West Africa/Outside African region	1	p = 0.250			1	
	East/Central Africa	1.96	0.50–7.69			2.24	p = 0.382
							0.31–16.05
	Southern Africa	4.06	0.70–23.66			6.76	p = 0.040*
							1.11–2.67
Highest qualification	Reference: None/Primary			1			
	Secondary			4.12	p = 0.057		
					0.95–17.87		
	Post-secondary/Diploma/Higher Education			6.23	p = 0.036* 1.16–33.48		
Number of years living in South Africa		1.16	p = 0.168 0.93–1.45	1.04	p = 0.188 0.98–1.11		
Type of facility visited	Reference: Central/Regional Tertiary hospital	1	p = 0.563			1	p = 0.392
	District/Regional hospital	0.73	0.07–7.39	0.32	p = 0.270 0.04–2.77	0.24	0.03–3.01
	PHC facility	0.44	0.09–2.20	0.20	p = 0.018* 0.05–0.71	0.53	0.09–5.18

OR = Odds ratio; CI = confidence interval. Only variables significant at a 20% level were included in the multivariate logistic regression model; *Significance = p < 0.05

**Table 2b.** Multivariate logistic regression model of socio-demographic and facility factors influencing patients’ perceptions of responsiveness (by domains: confidentiality and dignity)

		Domain: Confidentiality		Domain: Dignity	
		Do you think your privacy was respected?		Were you treated politely?	
Variable		OR	p-value/CI	OR	p-value/CI
Age		1.06	p = 0.144	1.08	p = 0.100
			0.97–1.16		0.98–1.20
Place of birth	Reference: North Africa/West Africa/Other			1	
	East/Central Africa			2.09	p = 0.426
					0.29–15.25
	Southern Africa			5.06	p = 0.027*
					1.26–20.32
Number of years living in South Africa					
Type of facility visited	Reference: Central/Regional Tertiary hospital			1	p = 0.08
	District/Regional hospital			1.16	0.16–8.22
	PHC facility			0.30	0.08–1.12

OR = Odds ratio; CI = confidence interval. Only variables significant at a 20% level were included in the multivariate logistic regression model; *Significance = p < 0.05

**Table 2c.** Multivariate ordinal logistic regression model of socio-demographic and facility factors influencing patients’ perceptions of responsiveness (by domain: communication)

		Domain: Prompt attention	
		Would you say the amount of time you spent for your visit was: too much; just right; too short?	
Variable		OR	p-value/CI
Place of birth	Reference: North Africa/West Africa	1	p = 0.194
	East/Central Africa	0.45	0.09–2.33
	Southern Africa	0.60	0.09–3.71
Type of facility visited	Reference: Central/Regional Tertiary hospital	1	p = 0.925
	District/Regional hospital	0.72	0.08–6.26
	PHC facility	0.80	0.22–2.91

OR = Odds ratio; CI = confidence interval. Only variables significant at a 20% level were included in the multivariate ordinal logistic regression model; *Significance = p < 0.05

**Table 2d.** Multivariate logistic regression model of socio-demographic and facility factors influencing patients’ perceptions of responsiveness (by domain: quality)

			Domain: Quality			
			Were you given any of the medicines prescribed for you?		If your friend or relative was sick would you encourage then to come to this clinic or hospital?	
Variable			OR	p-value/CI	OR	p-value/CI
Gender		Reference: Male			1	p = 0.034*
		Female			0.34	0.13–0.90
Age			1.02	p = 0.319	1.03	p = 0.053
				0.97–1.08		0.10–1.07
Place of birth		Reference: North Africa/West Africa			1	p = 0.098
		East/Central Africa			0.38	0.04–3.95
		Southern Africa			1.67	0.16–17.94
Highest qualification		Reference: None/Primary	1	p = 0.053		
		Secondary	3.01	0.98–9.21		
		Post-secondary/Diploma/Higher	5.74	p = 0.031*		
				1.21–27.18		
Number of years living in South Africa			1.02	p = 0.297		
				0.97–1.08		
Type of facility visited	Reference: Central/Regional Tertiary hospital		1	p = 0.904	1	p = 0.414
	District/Regional hospital		1.32	0.33–5.29	2.44	0.45–13.29
	PHC facility		1.03	0.40–2.66	0.75	0.26–2.15

OR = Odds ratio; CI = confidence interval. Only variables significant at a 20% level were included in the multivariate logistic regression model; *Significance = p < 0.05

#### Domain: communication

In this domain, a multiple logistic regression model was used to investigate factors associated with whether or not the nurse or doctor listened. The binary response variable was whether (1) or not (0) the nurse or doctor listened. The potential explanatory variables were age, place of birth, highest qualification, number of years living in South Africa, and type of facility visited. Education and type of health facility influenced whether patients knew the name of the attending clinician (nurse or doctor). The odds of knowing the name of the attending clinician were significantly higher for those with post-secondary education (OR = 6.23; 95% CI 1.16–33.48; p = 0.036). Similarly, patients from Southern Africa were 6.76 more likely to have received information about their condition (95% CI 1.11–2.67; p = 0.040). In contrast, patients at PHC facilities were less likely to know the name of the attending nurse or doctor (OR = 0.20; 95% CI 0.05–0.71; p = 0.018) (Table 2(a)).

#### Domain: dignity

In the domain of dignity, a multiple logistic regression model was used to explore the factors associated with patients reporting whether they had been treated politely or not. The binary response variable was whether (1) or not (0) patients reported being treated politely. The potential explanatory variables were age, place of birth, number of years living in South Africa and type of facility visited. Patients from Southern Africa had a higher odds of reporting that they have been treated politely (Table 2(b)), compared to patients from North Africa/West Africa/Other (OR = 5.06; 95% CI 1.26–20.32; p = 0.027).

#### Domain: prompt attention

We did not find any variables that were associated with patients’ perceptions of the waiting times at the health facility (Table 2(c)).

#### Domain: quality

In this domain, a multiple logistic regression model was used to explore the factors associated with firstly, whether patients had been given their prescribed medicines and secondly, whether patients would encourage a sick friend or relative to come to the clinic or hospital. The binary response for both variables were (1) yes or (0) no. The potential explanatory variables for whether patients had been given their prescribed medicines were gender, age, place of birth, highest qualification, number of years living in South Africa and the type of facility visited. Compared with those with no or primary education, patients with post-secondary, diploma or higher education were 5.74 times more likely (95% CI 1.21–27.18; p = 0.031) to indicate that they received medication (Table 2(d)).

The potential explanatory variables for whether patients would encourage a sick friend or relative to come to the clinic or hospital were gender, age, place of birth, number of years living in South Africa, and type of facility visited. Female patients were less likely to refer a sick friend or relative to the health facility (OR = 0.34 95% CI 0.13–0.90; p = 0.034).

## Relationship between HSR and patient satisfaction with health workers

Patients who were treated politely were 4.79 times more likely to be satisfied with clerks (95% CI 1.81–2.11). Similarly, patients who would refer the facility to a family member or friend were 1.50 times more likely to express greater satisfaction with clerks compared to those who would not refer (95% CI 1.00–2.24).

Patients who reported that time spent at the facility were shorter than expected, were 4.79 times more likely to express satisfaction with doctors (95% CI 0.44–16.00). Similarly, patients who reported that time spent at the facility were just right, were 4.15 times more likely to express satisfaction with doctors (95% CI 0.72–10.01), compared to patients who indicated that time spent at the facility were too long.

The odds of patient satisfaction with nurses were higher for those patients who reported that they received information about their condition compared to those who did not (OR = 2.68; 95% CI 1.45–4.97). Likewise, the odds of increased satisfaction with nurses were four times higher for patients who were treated politely (OR = 4.91; 95% CI 1.68–14.33).

Patients who indicated time spent at the facility was just right (OR = 2.53; 95% CI 1.53–4.18) or shorter than expected (OR = 2.70; 95% CI 1.22–5.96) had higher odds of reporting increased satisfaction with nurses, compared to those who reported that time spent at the facility was too much. Similarly, the odds of satisfaction with nurses were higher for patients who received their prescribed medicines (OR = 1.74; 95% CI 1.01–2.98). Likewise, patients who would refer the facility to a sick family member or friend had higher odds of expressing satisfaction with nurses compared to those who would not (OR = 2.38; 95% CI1.43–3.96). Table 3 shows the association between the HSR variables and patient satisfaction with health workers.

**Table 3.** Multiple ordinal logistic regression model of HSR and patient satisfaction with health workers

		Category of health care worker							
		Clerks		Security guard		Doctor		Nurse	
Variable		OR	Confidence Interval	OR	Confidence Interval	OR	Confidence Interval	OR	Confidence Interval
Communication	Do you think the nurse/doctor listened to you? (Reference: no)					3.39	0.27–42.53		
	Were you given information about your condition? (Reference: no)					0.34	0.09–1.27	2.68	1.45–4.97*
Confidentiality	Do you think your privacy was respected? (Reference: no)					0.86	0.15–4.88	1.32	0.19–9.01
Dignity	Were you treated politely? (Reference: no)	4.79	1.81–2.11*			16.15	0.25–1032.20	4.91	1.68–14.33*
Prompt attention	Would you say the amount of time you spent for your visit was:								
	Too Much (reference)					1		1	
	Just Right					4.15	0.72–10.01*	2.53	1.53–4.18*
	Too Short					4.79	0.44–16.00*	2.70	1.22–5.96*
Quality	Were you given any of the medicines prescribed for you? (Reference: no)							1.74	1.01–2.98*
	If your friend or relative was sick would you encourage then to come to this clinic or hospital? (Reference: no)	1.50	1.00–2.24*	2.06	0.62–6.77	0.83	0.24–2.89	2.38	1.43–3.96*

OR = Odds ratio; CI = confidence interval. Only variables significant at a 20% level were included in the multivariate ordinal logistic regression model; *Significance = p < 0.05

## Qualitative themes

Although overlapping, four themes emerged from the qualitative comments: health workers’ attitudes; time waited at the health facility, communication difficulties during consultation; and sub-optimal procedures in the health facility.

### Health workers’ attitudes

Patients’ comments on the attitudes of health workers were both positive and negative aspects. Their positive comments included friendliness or politeness, and ‘good’ attitudes or treatment.

*“I was treated very well, even health providers were friendly”* (Male, Zimbabwe, Specialised hospital)

“*Good. Staff received me well and all the things went well*.” (Female, Ivory Coast, Clinic)

In contrast, some patients described poor, uncaring attitudes, lack of sympathy, and sub-optimal treatment from health workers. Some patients highlighted the labour ward and emergency unit as problematic spaces for health care.

“*I didn’t like their treatment. I was robbed and injured and expected some sympathy and care”* (Male, Zimbabwe, District hospital)

“*First I would tell them to treat people nicely in emergency. When I bring the baby to casualty, they say why I don’t go to the clinic. But [the] clinic is closed. They don’t talk nice to me. Many nurses from here are not nice but doctors are nice*” (Female, Tanzania, Specialised hospital)

Some patient comments highlighted the intersection between nationality or being foreign, poor treatment, negative attitudes, and language difficulties.

“*They are rude especially to foreigners who do not understand the language* … ” (Female, Mozambique, Clinic)

“*As a foreigner I feel that nurses discriminate because of nationality*” (Female, Zimbabwe, CHC)

### Patients’ experiences of time spent at the health facility

Some patients voiced their dissatisfaction about the long time spent at the health facility, having arrived the previous day, and returning on the day of the fieldwork, while others linked long waiting times to ‘slow’ staff.

“I*t was bad, waiting since yesterday until now. We got here at 11.30am*.” (Female, Malawi, Regional hospital)

“*I have been here since last night, got injury on my leg only to be attended now, hungry and pains*” (Male, Somalia, Central hospital)

“*Bad, many people were even complaining saying that the staff is very slow today which lead to longer waiting times*.” (Male, DRC, Central hospital)

One patient explained that although she waited four hours for assistance, she was happy with the service she received.

“*I arrived at 6 and at about 10 am, I was done which is good. I have no complaints*” (Female, Malawi, CHC)

### Communication difficulties during health care consultations

Some patients reported the problems of poor or absent communication and information, exacerbated by language differences.

“T*hings are mixed up. They* [health workers] *don’t know who is coming for what. Staff need to provide better information to patients”* (Female, DRC, District hospital)

“*Difficult to understand the language”* (Male, Burundi, Clinic)

### Sub-optimal procedures in health facility

In this theme, patients highlighted the deficiencies that existed within the health facility, such as the administrative burden or procedures, constant administrative changes, as well as a general lack of organisation.

“*There is too much administration and it leads to longer waiting hours. [I] had a referral letter from the clinic but was sent to the gateway clinic and back which was unnecessary and strenuous for us and our sick baby*” (Female, Zimbabwe, District hospital)

“*Not well organized. Every month you come, things are different. Things change every month*.” (Female, Nigeria, District hospital)

## Discussion

This study aimed to examine the perceptions of migrants on health system responsiveness (HSR) and their satisfaction with health workers in public health facilities. We also assessed the relationships between socio-demographic and health facility characteristics and HSR, and the association between HSR and patient satisfaction with health workers.

In 2018, the demographic profile of migrant patients utilising public health facilities in the Gauteng Province of South Africa were female (82.0%), and the mean age was 31.5 years. In this study, 55.7% of patients surveyed were from Zimbabwe, the northern neighbour of South Africa. This finding is not surprising, given the proximity of Zimbabwe to South Africa, and the virtual collapse of the health system in that country [41].

Although 54.6% of patients reported that the amount of time for their visit was ‘just right’, almost one-third (30.0%) complained about long time that they spent at the health facility. Complaints about waiting times also emerged in the qualitative comments, with some patients highlighting that they arrived the previous day, but had to return, and wait again. We could not find other comparable studies in South Africa that surveyed migrant patients about their perceptions of waiting times. However, the figure of 30.0% of migrant patients who felt that the waiting times were too long is similar to the findings of the 2010 General Household Survey, showing that 34.8% of South African adults reported that long waiting times were the most common problem experienced during their most recent visit to a healthcare provider [42]. These findings suggest that waiting times are a problem for all individuals utilising public health services. Our study findings are supported by the 2016/17 inspection report of the Office of Health Standards Compliance (OHSC) that found that Gauteng had the longest waiting times when compared to other provinces [43].

A possible explanation for the majority (55.6%) of migrant patients’ apparent satisfaction with waiting times could be because of their prior expectations (whether in their home country or South Africa) that waiting times would be longer than the actual time spent at health facilities. Although the context is different, the influence of prior expectations on patient satisfaction with waiting times was also found in a Canadian study [44]. In the regression model, we could not find any variables that were associated with migrant patients’ perceptions of waiting times. However, a qualitative study in the USA found that patients’ ‘willingness to wait’ was influenced by the actual wait time, the perceived value of the visit, the cost of waiting, and health facility and provider factors [45]. Hence, this area needs further research in South Africa.

Although the majority of patients (94.3%) indicated that the health care worker listened to them and that they received information about their condition (89.4%), the qualitative comments revealed the problems of insufficient information and inadequate communication about health service delivery. Patients also complained that they did not understand the language spoken. Other South African studies show that some health care providers refuse to speak in English, making it difficult for migrant patients to understand them [46,47]. Language barriers in the health care setting are a common problem found in studies with migrant patients in the UK and Germany [17,48].

In our study, 81.7% of patients reported that they did not know the name of the attending nurse or doctor. However, those migrant patients with secondary and post-secondary education were more likely to know the name of the attending health provider. This could be because those patients with secondary or post-secondary education noticed and remembered the name badges of health care providers, as it is one of the quality of care standards in South Africa [49]. It could also be that patients with higher education levels ensured mutual introduction between themselves and the attending health provider.

We also found that patients at PHC facilities were significantly less likely to know the name of the attending health care provider. This could be because health workers do not wear name tags, an area that the OHSC flagged for improvement in its 2016/2017 inspection report of the Gauteng Province [43].

The vast majority of patients indicated that they were treated politely. Patients from Southern Africa were more likely to indicate that they were treated politely during their visit to the health facility. This is surprising and could relate to prior expectations or past experiences in South Africa [45], the resource constraints of the health systems in their home countries [41], and/or the similarities of indigenous languages in these countries, with those in South Africa.

The majority of participants (80.6%) reported that they received their prescribed medication. Those with secondary or post-secondary education were more likely to indicate receiving their prescribed medicines. We could not find other studies demonstrating a relationship between education levels and receipt of prescribed medicines. However, there is global evidence of the positive correlation between higher levels of education, health and well-being, and utilization of preventive services [50,51]. It could be that those migrant patients with more years of education were more likely to pay attention to medicines prescribed, request medication from the provider, and/or ensure that they received prescribed medication.

Although the majority of patients said that they would refer a sick friend or family member to the facility, female patients were less likely to refer the facility to a family member or friend. This could be because health service utilisation among women tends to be higher than among men [52–54], and the majority of patients in this study were women. Thus, their experiences of the facility may have influenced their responses on whether they would refer friends or family members.

Clerks received the lowest satisfaction mean score of 7.0. This could be because clerks are often the gatekeepers to clinical care services in health facilities, because they register patients and prepare the medical files. Those patients who reported that they received polite treatment were more likely to indicate satisfaction with clerical services.

Nurses received a lower patient satisfaction mean score of 7.4, compared to doctors who received the highest satisfaction mean score of 8.3. In part, this is because of the numerical dominance of nurses, who are the category of health workers that patients are more likely to encounter. Although some patients commented positively on nurses, there were also complaints about nurses’ uncaring attitudes and perceptions of discrimination. Similar complaints on the uncaring attitudes of nurses were also described in the 2010 Consolidated Report on Inspections of Primary Health Care Delivery Sites in Gauteng [55]. Hence, the health minister declared ‘values and attitudes’ as a priority for action and monitoring. Although the OHSC inspection report for 2016/17 found that Gauteng Province obtained the highest score of 68% for values and attitudes, the OHSC highlighted that this priority area needs ongoing attention [43].

The patient complaints about discrimination because of nationality are of concern, and have been found in other studies as well [21,56]. We did not ask patients explicitly whether they experienced discrimination, and this needs further research. Nonetheless, discrimination is unacceptable, and all health managers should put in place systems to prevent discrimination, and to ensure that health workers uphold their ethical obligations. In this study, patients who indicated that they were treated politely, given information about their condition, received prescribed medicines and would refer the health facility to family and friends were more likely to express higher satisfaction with nurses. This suggests a close relationship between perceptions of HSR and satisfaction with health workers.

Our study found that those patients who indicated that the time spent in the health facility were just right or shorter than expected, were more likely to express satisfaction with the attending nurse or doctor. A 2017 South African study on patient satisfaction with nurse PHC delivery also demonstrated a positive relationship between shorter waiting times, and patient satisfaction with nursing care [57]. Although not comparable because of context and variations in measurement and study design, studies from China, OECD countries and the USA have found inverse relationships between waiting times and patient satisfaction [58–61]. Hence, the reduction of waiting times for all patients remains a priority in the South African public health system.

Our study is limited by its cross-sectional nature, as it represents the views of migrant patients at a point in time. Another limitation is that we conducted the study in one South African Province, which means that the study is not generalisable to the entire country. We conducted our study in English, which is a second language for many migrants, and this might have constrained their ability to comment extensively. Our study surveyed migrant patients utilising services at the health care facilities, rather than examine perceived access barriers. Hence, our findings are a reflection of these patients already in the health care system, and may not be generalizable to all migrants. Lastly, although we reassured participating patients of the independence of the research team, some responses may have been guarded. Although we included a range of socio-demographics, we did not include employment and level of income in our questionnaire – this is a study limitation and we would not be able to explore socio-economic status of those participants in our study.

However, there are numerous study strengths. This was one of the first comprehensive surveys that examined migrants’ perceptions of HSR, and their satisfaction with health workers. We obtained a high response rate among migrant patients, thus overcoming the potential bias of non-response. We selected the facilities and fieldwork days randomly, thus overcoming the potential problem of selection bias. The open-ended questions allowed study participants to narrate their experiences of the health facility visit in their own words, thus adding valuable insights into migrant patients’ experiences in Gauteng public health facilities.

The survey questionnaire can be used or adapted by other researchers to measure HSR and patient satisfaction, whether in South Africa, other LIMICs, or among other vulnerable groups of patients.

The gaps identified in our study such as perceived long waiting times, sub-optimal communication, and uncaring attitudes of frontline health workers have occupied the health policy reform agenda since democracy, and remain critical priorities to address [62]. The 2019 South African Lancet National Commission recommended improved leadership and governance, revolutionising quality of care; investment in the health workforce, and measurement, monitoring and evaluation to achieve of a high quality health system [62]. The implementation of these recommendations are likely to benefit all patients, regardless of nationality, in the South African public health system.

## Conclusion

This study examined migrants’ perceptions of HSR and their satisfaction with health workers. Our study illustrates that migrant patient satisfaction and their experiences are complex and nuanced. We found that perceptions of HSR are closely linked to satisfaction with health workers.

Further research should incorporate the factors that influence migrants’ access to care, compare the experiences of migrant patients to those of South Africans, and complement the HSR survey with in-depth qualitative studies over a longitudinal period.

HSR and patient satisfaction are important elements of quality UHC. However, vulnerable groups, such as migrants, are at risk of exclusion from the health care systems of their host countries. In South Africa, as elsewhere, quality UHC is a priority. Our study findings should be incorporated into the design of inclusive UHC policies, so that all patients, regardless of nationality, can benefit from high-quality public health systems.

## Data Availability

This data is part of a doctoral study, which will be examined in 2020. The ethics number is M170988. All patients gave their consent for confidential participation before participating in the survey. No personal information was collected. Data access is limited for ethical reasons to controlled access within the Redcap server. The data is available to any Bona fide researchers with appropriate ethics clearance from their institution or on application to the University of Witwatersrand Human Research Ethics Committee (Medical). However, all data is available outside of the Redcap server to accredited peer reviewers on a specific peer review access.

## Paper context

Within the global context of achieving high-quality universal health coverage (UHC), the world faces an unprecedented migrant crisis. This study generated new knowledge on migrant patients’ perceptions of health system responsiveness (HSR) and their satisfaction with health workers in public health facilities of a South African Province, as well as the factors that influence these perceptions. The study findings have implications for the design of inclusive UHC policies.
